# Efficacy of indoor residual spraying with broflanilide (TENEBENAL), a novel meta-diamide insecticide, against pyrethroid-resistant anopheline vectors in northern Tanzania: An experimental hut trial

**DOI:** 10.1371/journal.pone.0248026

**Published:** 2021-03-03

**Authors:** Janneke Snetselaar, Mark W. Rowland, Baltazari J. Manunda, Ezekia M. Kisengwa, Graham J. Small, David J. Malone, Franklin W. Mosha, Matthew J. Kirby

**Affiliations:** 1 London School of Hygiene & Tropical Medicine, London, United Kingdom; 2 Kilimanjaro Christian Medical University College, Moshi, Kilimanjaro, Tanzania; 3 Innovative Vector Control Consortium, Liverpool, United Kingdom; University of Crete, GREECE

## Abstract

Novel chemistry for vector control is urgently needed to counter insecticide resistance in mosquitoes. Here a new meta-diamide insecticide, broflanilide (TENEBENAL^TM^), was evaluated in East African experimental huts in Moshi, northern Tanzania. Two consecutive experimental hut trials with broflanilide 50WP were conducted; the first evaluating the efficacy of three concentrations, 50 mg/m^2^, 100 mg/m^2^, and 200 mg/m^2^ using a prototype formulation, and the second trial evaluating an improved formulation. The IRS treatments were applied on both mud and concrete surfaces and efficacy was monitored over time. The mortality, blood-feeding inhibition and exiting behaviour of free-flying wild mosquitoes was compared between treatment arms. Additionally, cone assays with pyrethroid-susceptible and resistant mosquito strains were conducted in the huts to determine residual efficacy. The first trial showed a dosage-mortality response of the prototype formulation and 3–8 months of residual activity, with longer activity on concrete than mud. The second trial with an improved formulation showed prolonged residual efficacy of the 100 mg/m^2^ concentration to 5–6 months on mud, and mosquito mortality on the concrete surface ranged between 94–100% for the full duration of the trial. In both trials, results with free-flying, wild *Anopheles arabiensis* echoed the mortality trend shown in cone assays, with the highest dose inducing the highest mortality and the improved formulation showing increased mortality rates. No blood-feeding inhibition or insecticide-induced exiting effects were observed with broflanilide. Broflanilide 50WP was effective against both susceptible and pyrethroid-resistant mosquito strains, demonstrating an absence of cross resistance between broflanilide and pyrethroids. The improved formulation, which has now been branded VECTRON^TM^ T500, resulted in a prolonged residual efficacy. These results indicate the potential of this insecticide as an addition to the arsenal of IRS products needed to maintain both control of malaria and resistance management of malaria-transmitting mosquitoes.

## Introduction

Control of malaria-transmitting mosquitoes is highly dependent on insecticidal products, mainly Indoor Residual Sprays (IRS) and Insecticide Treated Nets (ITNs). Widespread deployment of these vector control measures has drastically reduced malaria incidence and mortality in Sub-Saharan African in the last 20 years [1–3]. It was estimated that between 2000 and 2015 approximately 10% of the reduction in malaria cases was due to the use of IRS [2]. However resistance to pyrethroids on the African continent has spread rapidly and is threatening the gains made in decreasing the malaria burden [4, 5]. Between 2009 and 2013 many IRS campaigns shifted from using pyrethroids products to using alternative insecticides to counter malaria being transmitted by pyrethroid resistant vectors.

The importance of IRS has recently been reiterated by WHO as a core intervention for malaria control [6]. The optimal deployment of IRS is by rotation in time or in spatial mosaics (rotation by area) using multiple products with no common mode of action, to restrict exposure to any single insecticide class [7]. To delay the emergence and spread of insecticide resistance, the WHO recommends rotation of at least three insecticides that target different sites in the mosquito. To be able to spray insecticides in rotation or mosaic designs, new classes of insecticides are needed to sustain efforts towards malaria elimination over the next 20 years [7–9]. Currently most IRS operational strategies on the African continent employ either an organophosphate or a neonicotinoid product. The organophosphate Actellic® 300CS is a capsule suspension formulation of pirimiphos-methyl (PMM) [10] that attained a recommendation for use by the WHO Pesticide Evaluation Scheme (WHOPES) in 2013 [11]. In recent years two neonicotinoid formulations for IRS were listed by the WHO prequalification team vector control (WHO-PQT VC) [12]. Both these insecticide formulations contain the novel public health active ingredient clothianidin, either in a wettable granule formulation, SumiShield® 50WG, or in combination with deltamethrin as a wettable powder, Fludora® Fusion. An IRS formulation containing a third novel insecticide, with a distinct mode of action, that could be used in future rotation for insecticide resistance management (IRM) [8], was tested in the studies described here.

Broflanilide *N*-[2-bromo-4-(perfluoropropan-2-yl)-6-(trifluoromethyl)phenyl]-2-fluoro-3-(*N*-methylbenzamido)benzamide] is a novel active ingredient developed by Mitsui Chemicals Agro Inc. (MCAG; Tokyo, Japan) [13] that has potential for use in vector control, including the control of mosquitoes which have become resistant to pyrethroids and potentially other classes of insecticide [14]. Broflanilide (tradename TENEBENAL^TM^) is a meta-diamide insecticide that has a different mode of action compared to all other insecticides currently used in public health [15]. The active ingredient targets the GABA-receptor in the nervous system of mosquitoes, and has been classified by the Insecticide Resistance Action Committee (IRAC) in the new class 30; GABA-gated chloride channel allosteric modulators [16]. The development of broflanilide 50WP in a vector control product was coordinated between the London School for Health and Tropical Medicine (LSHTM), the Kilimanjaro Christian Medical University College (KCMUCo), MCAG and the Innovative Vector Control Consortium (IVCC).

Before bringing novel chemistry to market for vector control, decisions need to be made on the optimal dose and formulation for the active ingredient. Previous studies have shown that the formulation of an insecticide can have a significant impact on its residual efficacy [17, 18]. Ideally a combination of formulation, concentration and application rate is selected that optimises safety, cost-effectiveness and duration of effective action against the target insect [19]. IRS programmes should utilise insecticides that have a residual activity sufficient so that a single application is effective for the entire malaria transmission season. In many sub-Saharan African countries this equates to a duration of effective action of 6 months or longer. In areas with a single transmission season, 6 months effectiveness is sufficient to provide protection, but a longer residual efficacy is desirable in areas with two rainy seasons or year-round transmission [20]. This study presents the first experimental hut evaluations of broflanilide 50WP in East Africa. Two trials were conducted: The first trial investigated the residual efficacy of three different concentrations of a prototype formulation (B2) on concrete and mud substrates; the second trial compared two formulations of broflanilide, B2 and B3, with the latter developed specifically to improve the residual efficacy of the insecticide on mud surfaces.

## Methodology

### Study location and mosquitoes

This study was conducted at the Harusini field station (3°40’S, 37°36’E) of the OECD GLP-certified KCMUCo-PAMVERC Test Facility (G0023, SANAS) located in the Moshi rural district in the Kilimanjaro region, Tanzania. The dominant malaria vector species in this area is *Anopheles arabiensis*, which breeds in the irrigated rice fields surrounding the field station [21]. Mosquito numbers peak during the early stages of the rice growing cycle, when the rice fields are flooded and rice seedlings are planted. The mosquito season typically lasts for 6–8 weeks in each rice cycle and on average two cropping cycles are farmed per year.

Cone assays using insectary-reared mosquitoes were conducted each month, independent of the mosquito season. Two insectary-reared mosquito strains were used; the susceptible *An*. *gambiae* s.s. Kisumu strain and the pyrethroid-resistant *An*. *gambiae* s.s. Muleba-Kis strain. The Kisumu strain is a standard insecticide susceptible strain lacking the *kdr* alleles (L1014S and L1014F) coding for pyrethroid resistance. The Muleba-Kis strain was colonised in 2012 from eggs from Muleba, north-west Tanzania, an area where the *An*. *gambiae* s.s. wild population showed phenotypic resistance to pyrethroids and a frequency of the *kdr* east mutation near fixation [22]. The Muleba-Kis strain tested 100% homozygous for the L1014S mutation and in analysis of P450 enzymes associated with metabolic resistance, CYP6P3 was found to be overexpressed. Routine characterisation of phenotypic resistance using WHO test papers [23] indicated susceptibility to carbamates and organophosphates for both strains, and resistance of the Muleba-Kis strain to organochlorines and pyrethroids. The Ace-1 (G119S) mutation is absent from both strains. Increased transcription of the P450 gene CYP4G16, associated with permethrin resistance, was found for the wild *An*. *arabiensis* population [24]. CDC bottle bioassays conducted in 2019 showed 5x the diagnostic concentration of α-cypermethrin killed 82% of mosquitoes, increasing to 100% mortality with pre-exposure to piperonyl butoxide (PBO). This is further evidence of metabolic resistance in this wild population.

### Treatments

The two formulations under evaluation were 50% wettable powder formulations containing broflanilide (tradename TENEBENAL^TM^). Three application rates of the protype formulation (B2; batch no. 17I-3422) were used in the first experimental hut trial. The second trial included the improved formulation B3 (batch no 18I-3671) applied at two different rates and compared to formulation B2 (batch no 16I-3147) (Table 1).

**Table 1:** Experimental treatments of the two experimental hut trials.

Spray date	Hut no.	Active ingredient	Formulation	Concentration mg/m^2^	Substrate
Sep 2017	6	-	-	-	concrete
	3	broflanilide	WP (B2)	50	concrete
	8	broflanilide	WP (B2)	100	concrete
	5	broflanilide	WP (B2)	200	concrete
	7	pirimiphos methyl	CS	1000	concrete
	2	broflanilide	WP (B2)	50	mud
	4	broflanilide	WP (B2)	100	mud
	1	broflanilide	WP (B2)	200	mud
July 2018	2	-	-	-	concrete
	7	broflanilide	WP (B2)	100	concrete
	6	broflanilide	WP (B3)	100	concrete
	4	broflanilide	WP (B3)	150	concrete
	8	pirimiphos methyl	CS	1000	concrete
	5	broflanilide	WP (B2)	100	mud
	1	broflanilide	WP (B3)	100	mud
	3	broflanilide	WP (B3)	150	mud

Three concentrations of the broflanilide wettable powder (WP) were compared in the first hut trial, and two prototype formulations (B2 and B3) were compared in the second experimental hut trial. In both trials pirimiphos methyl CS was used as a positive control.

Treatments were sprayed using manual compression sprayers, model CS-14 (Micron Sprayers Ltd, Bromyard, United Kingdom), with an 8002E nozzle spraying at an application rate of 30mL/m^2^ using a 1.5 bar constant flow valve (CFV). Each treatment was sprayed on both concrete and mud substrates: the walls and ceiling of each hut were treated. For both trials the reference product was an IRS formulation of the organophosphate insecticide pirimiphos methyl (Actellic®300CS, Syngenta®, Basel, Switzerland) applied at the recommended label rate of 1000 mg/m^2^, and the negative control was an untreated concrete-lined hut. Treatments were randomly allocated to the eight huts using the MS Excel RAND() function.

### Experimental huts

The experimental huts were of the East African design first described by Smith [25]. These have four verandahs, an eave gap [26] with eave baffles [27], and a water-filled moat to keep ants out. The mud surface was made of a soil:sand ratio of 4:7 and the concrete surface was made of a cement:sand ratio of 1:3. The concrete surface was allowed to cure for 4 weeks until the pH of the concrete was within an acceptable range before spraying. The ceiling of each hut was composed of the same substrate as the walls. Spray tanks were allocated to specified active ingredients (a.i.) and formulations to avoid any risk of cross contamination. All spray tanks were calibrated prior to spraying following WHO methodology [28]. 70cm spray swaths with a 5cm overlap were marked out on the walls and ceilings of huts to aid the spray operator. Hut walls measured 278cm wide and 186cm high, resulting in a total sprayable surface of 28.41m^2^ per hut. Average flow rate was calculated prior to spraying for each spray tank and accepted when the flow rate was 550±10% ml/min. Spray operators were trained according to GLP guidelines.

### Quality control of IRS application

Spray operators were extensively trained on standard operating procedure (SOPs) detailing lance speed, accurate distance from the wall, and movement during spraying. Spray tanks were calibrated and maintained according manufacturer’s instructions, and all phases of the spraying were supervised and controlled. As a quality control measure, the volume of left-over insecticide was measured post-spraying and used to calculate the volume of applied insecticide for each hut. Insecticide concentration was measured using two methods: extraction from liquid samples from the spray tanks, and extraction from filter papers mounted on the hut walls. Samples were analysed using High Performance Liquid Chromatography (HPLC) at the Liverpool School for Tropical Medicine (LSTM). The liquid samples were taken directly from the spray tank both before and after spraying huts. For each sample three replicates were extracted using a DCP (dicyclohexyl phthalate) in acetone extraction solution. The average active ingredient concentration in mg/mL was calculated for each sample.

Filter papers (9cm Ø; Whatman^TM^ no. 1) were attached to the walls and ceilings of experimental huts before the IRS spraying. Two filter papers were attached on each wall and two on the ceiling, giving a total of ten filter papers per hut. For the filter paper analysis twelve subsamples with a total surface area of 15.24 cm^2^ were punched out of the filter papers and the active ingredient was extracted using a DCP in acetone extraction solution. Samples were sonicated for 15 min and then dried until all acetone had evaporated. Prior to HPLC analysis samples were resuspended in 1 mL acetonitrile, centrifuged, and injected onto HPLC.

### Residual efficacy

Residual efficacy of the insecticidal treatments was evaluated using monthly cone assays as described in the WHO guidelines [29]. Two cones per wall and two on the ceiling were used in each round, with a total of 100 mosquitoes exposed in cones for each hut/month/strain. Cones were attached directly to the walls using masking tape; the location of each cone bioassay was randomised and marked on the wall using chalk to ensure that specific locations were not used more than once during each trial.

Mosquitoes were used in the assays as adult unfed females aged 2–5 days. After 30 minutes of exposure in cones the mosquitoes were maintained at ambient temperature and 75±15%RH in a holding room at the Harusini hut site. Knockdown was recorded at 60 min and mortality at 24h, 48h and 72h post-exposure. Cone bioassays were performed on the unsprayed concrete substrate in the control hut at the same time as cone bioassays in the treated huts.

### Wild collections

The insecticides were evaluated in the experimental huts against wild, free-flying mosquitoes which entered the huts naturally during the mosquito season, attracted by a cow that was restrained inside the huts. Cow-baited collection methods were previously shown to be more effective in catching the local mosquito vector *An*. *arabiensis* [30]. The cows selected were 1- or 2-year-old non-lactating calves of similar size and weight which did not receive any insecticide treatment before or during the trial. The treatment-hut association was fixed by position. To control for differences in attractiveness of individual cows to mosquitoes, cows were rotated daily between the huts using a Latin square design. Mosquito collection was done in the morning by two technicians. Dead mosquitoes were collected from inside the hut, the exit traps, and the verandahs. Live mosquitoes were captured from the exit traps and the verandahs only and brought to the holding room to monitor mortality over time up to 72h after collection. Live mosquitoes inside the hut were not captured but left to die inside or exit naturally into the traps. During both hut trials 80–100 wild type mosquitoes were packed individually into capsules, labelled with unique IDs, and analysed for species and *kdr* mutation L1014S.

### Molecular analysis

The species identification and presence/absence of *kdr* mutation L1014S was determined using Taqman real time assays for Anopheles species (3plex assay) [31] and knockdown resistance mutation (*kdr*) [32], respectively. Mosquitoes were identified as either Ar (*Anopheles arabiensis)* or Ga (*Anopheles gambiae s*.*s*.). For the resistance assays mosquitoes were classified as SSe, RSe or RRe i,e, homozygous susceptible, heterozygous, or homozygous resistant for the *kdr* mutation L1014S (*kdr*-East). Samples that did not amplify were re-run.

### Outcomes

The main outcome of the study was residual efficacy of the insecticide treatments by substrate, measured as mortality at 72h post-exposure for both insectary-reared mosquito strains. The period between the spray application and the last month mosquito mortality exceeded 80% was used as an estimation of duration of effective action of the insecticide. The 80% mortality threshold was introduced by WHOPES [29] for the evaluation of pyrethroid compounds. Mortality was evaluated at 72h post exposure rather than 24h in the present study because of the delayed mortality effect seen with broflanilide 50WP; 72h was the period post-exposure when the daily mortality rate of the treatment arm converged with that of the negative control arm.

For the wild free-flying mosquitoes the following outcome measures were assessed as described in the WHO guidelines [29]:

- The exit rate, which is the proportion of female mosquitoes found in the exit traps and verandas compared to the total number found in the hut. The difference in exit rate relative to the control was used to estimate induced exophily
- The blood feeding rate, which is the proportion of blood fed female mosquitoes compared to the total number found in the hut. The reduction of blood fed mosquitoes between a treated hut and the control hut was used to assess blood feeding inhibition due to the insecticide. Blood feeding inhibition was calculated as follows:

$$100*\frac{{B_{c}-B}_{t}}{B_{c}}$$

Where *B*_*c*_ is the percentage blood feeding in the control and *B*_*t*_ is the percentage blood feeding in the treatment.

- The mortality rate, which is the proportion of female mosquitoes found dead in the hut, on collection and 72h later. The difference in mortality between a control hut (natural mortality) and a treated hut was used to assess insecticide-induced mortality, calculated as follows:

$$100*\frac{{M_{c}-M}_{t}}{M_{c}}$$

Where *M*_*c*_ is the percentage mortality in the control and *M*_*t*_ is the mortality in the treatment.

### Statistical analysis

Data was entered into Microsoft Excel and statistical analysis were performed using Stata v.15 software (STATA Corp, College Station, USA). For the wild collections the proportion mosquitoes exiting the huts (exophily), the proportion blood feeding, and mortality of wild mosquitoes 72 hours after collection were compared using logistic regression for proportional data adjusting for the effects of cows. Treatments were significantly different at α = 0.05. Confidence intervals were adjusted for clustering per day.

Residual efficacy was determined using the average of ten cones with ten mosquitoes per strain and per treatment arm for each timepoint. For each substrate the insecticide was considered effective if mosquito mortality at 72h post-exposure was greater than the WHO 80% mortality threshold [29]. 95% confidence intervals were calculated using the following equation where $\hat{p}$ is the sample proportion, z is the confidence interval coefficient and n is the sample size, i.e. the total number of mosquitoes tested.

$$\hat{p}\pm z*\surd (\frac{\hat{p}(1-\hat{p})}{n})$$

### Ethics statement

The research programme was reviewed and approved by the National joint board of Ethics in Tanzania, comprising the Medical Research Coordinating Committee and the Ministry of Health, Community Development, Gender, Elderly & Children; certificate number NIMR/HQ/R.8a/Vol. IX/1646. The use of cows in experimental huts was reviewed by the Animal Welfare and Ethical Review Board (AWERB) and approval is given under LSHTM AWERB reference: 2020–02.

## Results

### Residual efficacy of broflanilide 50 WP against insectary-reared mosquito strains in experimental huts

**Fig 1:**
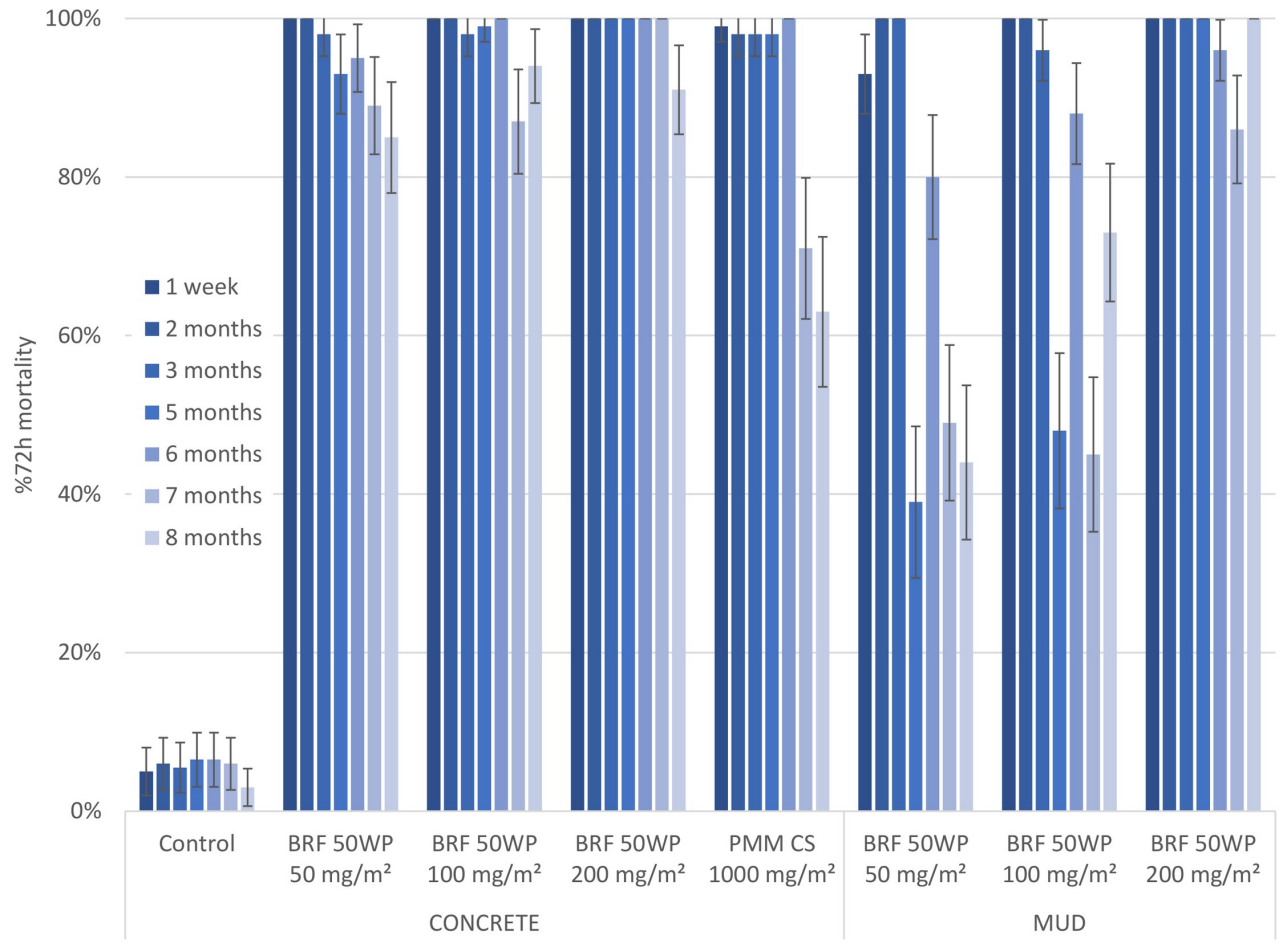
First hut trial; comparing three doses of broflanilide 50 WP. Residual efficacy against susceptible mosquitoes in cone assays. Residual efficacy of broflanilide 50 WP Recipe B2 (BRF) and pirimiphos methyl (PMM) sprayed on the walls and ceiling of experimental huts. Three concentrations of broflanilide 50 WP were tested over time. Cone assays with the susceptible An. gambiae s.s. Kisumu strain were conducted monthly and the mean 72-hour mortality ± 95% CI is given per time point, concentration and substrate.

In the first experimental hut trial, the residual efficacy of a prototype broflanilide 50WP formulation, B2, applied at three dosage rates was evaluated. Treatments were tested monthly for up to 8 months, apart from those months where insufficient mosquitoes were available. For the Kisumu strain broflanilide sprayed on a concrete surface met the WHO threshold of 80% mortality for 8 months (Fig 1). Mortality at 72 hours post-exposure ranged between 85% and 100% for all three application rates. The reference treatment, pirimiphos methyl (PMM), was also sprayed on concrete and mosquito mortality was between 98%-100% for 6 months and dropped to 71% and 63% mortality at month 7 and 8 respectively. On the mud surface, the efficacy of the 50 mg/m^2^ and 100 mg/m^2^ target doses of broflanilide dropped below 80% after 3 months and oscillated between ~40% and ~80% mortality for the remaining months. The highest application rate sprayed on the mud surface induced between 86% and 100% mortality for the whole trial, i.e. >8 months duration of effective action.

**Fig 2:**
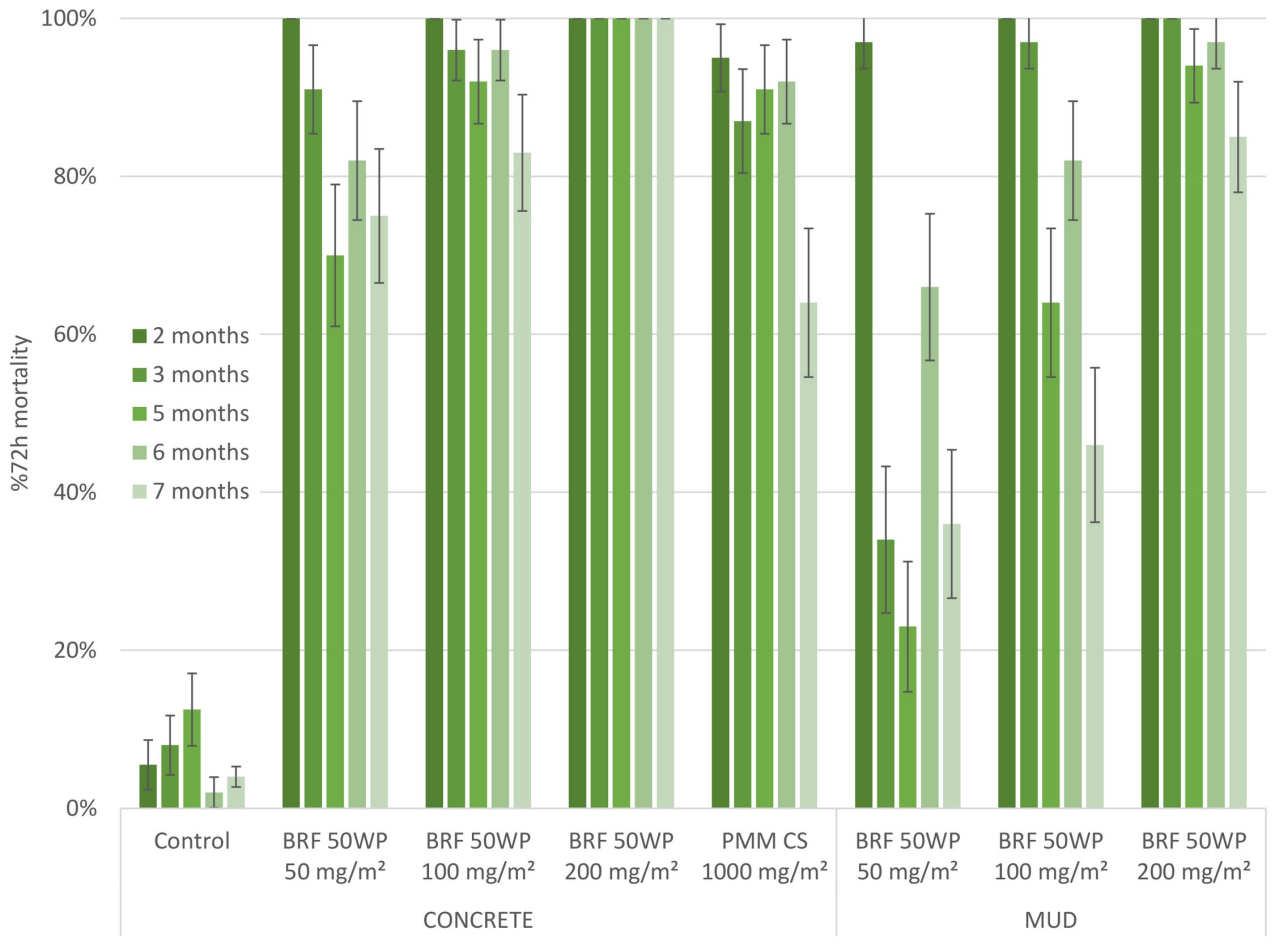
First hut trial; comparing three doses of broflanilide 50 WP. Residual efficacy against resistant mosquitoes in cone assays. Residual efficacy of broflanilide 50 WP Recipe B2 (BRF) and pirimiphos methyl (PMM) sprayed on the walls and ceiling of experimental huts. Three concentrations of broflanilide 50 WP were tested over time. Cone assays with the resistant An. gambiae s.s. Muleba-Kis strain were conducted monthly and the mean 72-hour mortality ± 95% CI is given per time point, concentration and substrate.

Monthly cone assays with the resistant Muleba-Kis strain showed similar results to the Kisumu strain (Fig 2), except that the lowest concentration of the broflanilide 50WP B2 formulation sprayed on concrete resulted in mortality levels marginally below the 80% threshold for month 5 (70.0%, 95% CI 61.0%-79.0%) and month 7 (75%, 95% CI 66.5%-83.5%). Otherwise, all applications sprayed on the concrete surface had a duration of effective action for the length of the trial, i.e. >7 months, whereas the PMM treatment remained efficacious for 6 months before mortality levels dropped to 64% at month 7. On the mud surface there was a clear dose response effect: the highest dose of the B2 formulation killed more than 80% of mosquitoes for the full duration of the trial whereas mortality with the 50 mg/m^2^ target dose fell below 80% at 2 months and likewise for the 100 mg/m^2^ target dose at 3 months after spraying.

**Fig 3:**
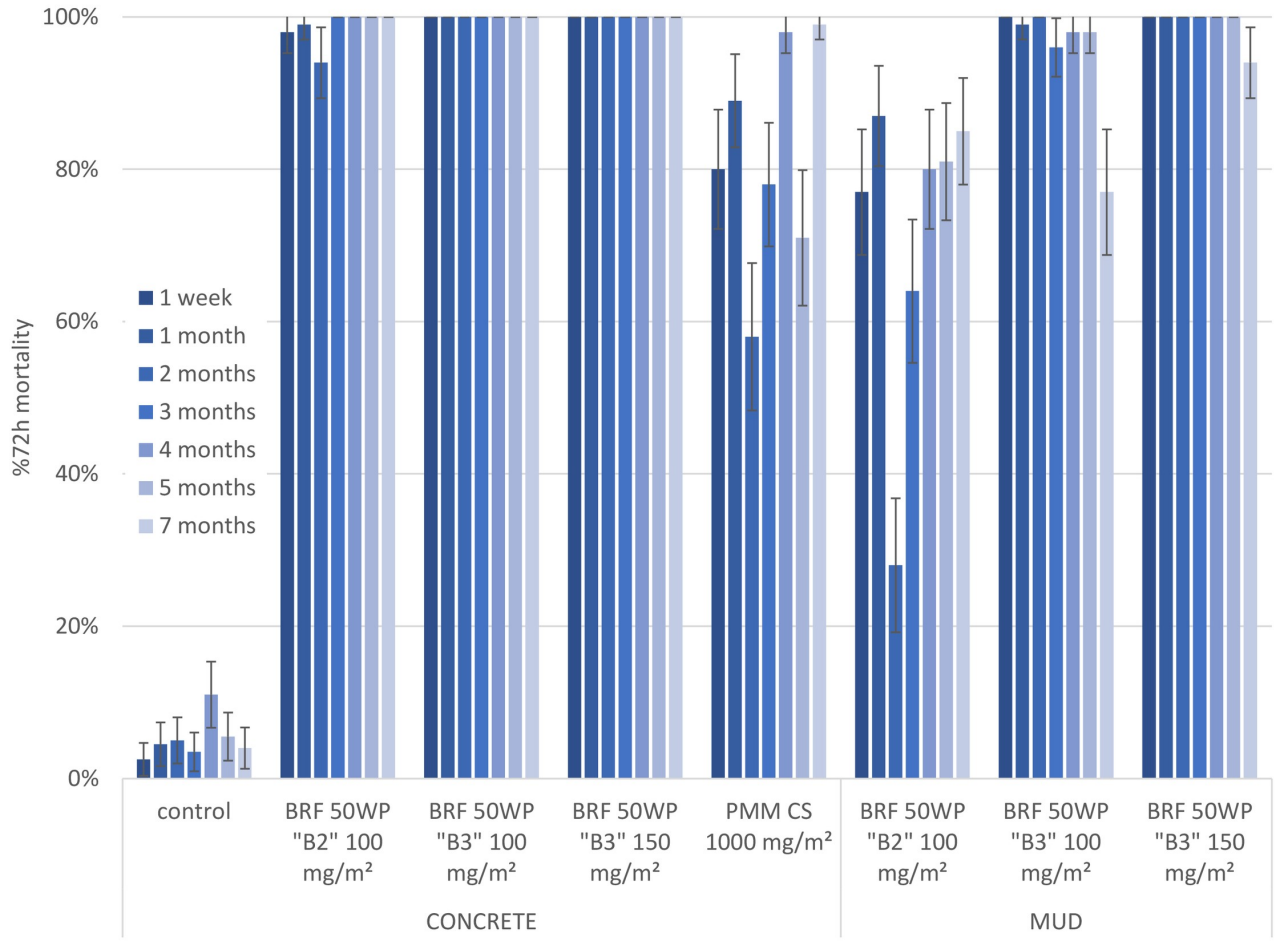
Second hut trial; comparing two formulations of broflanilide 50 WP. Residual efficacy against susceptible mosquitoes in cone assays. Evaluation of the B2 vs the B3 formulation of broflanilide 50 WP (BRF) and pirimiphos methyl (PMM). Residual efficacy was determined through monthly cone assays with susceptible An. gambiae s.s. Kisumu mosquitoes. For each time point, substrate and treatment tested the mean 72h mortality ± 95% CI is given.

**Fig 4:**
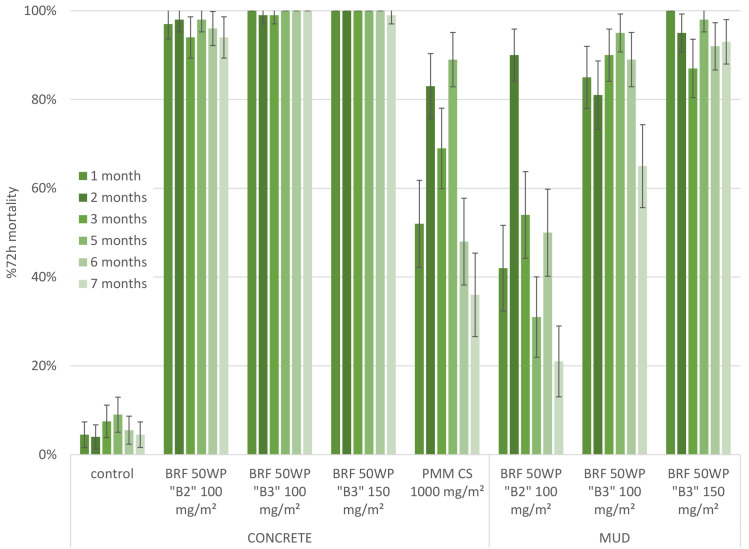
Second hut trial; comparing two formulations of broflanilide 50 WP. Residual efficacy against resistant mosquitoes in cone assays. Evaluation of the B2 vs the B3 formulation of broflanilide 50 WP (BRF) and pirimiphos methyl (PMM). Residual efficacy was determined through monthly cone assays with resistant An. gambiae s.s. Muleba-Kis mosquitoes. For each time point, substrate and treatment tested the mean 72h mortality ± 95% CI is given.

A second experimental hut trial was conducted with two concentrations of the improved formulation B3 of broflanilide versus the broflanilide 50 WP B2 formulation sprayed at a target dose of 100 mg/m^2^ (Figs 3 and 4). On the concrete surface treatments of both formulations gave mosquito mortality ranging between 94–100% for the full duration of the trial, i.e. 7 months, against both insectary-reared strains. The mean monthly mortality with the reference pirimiphos methyl product fluctuated over time, varying between 58% and 99% mortality for the Kisumu strain and between 36% and 93% mortality for the Muleba-Kis strain.

On the mud surface the B2 formulation induced mean mosquito mortality between 64% and 87% for Kisumu up to month 7, with the exception of month 2 where mortality was only 28%. For Muleba-Kis the efficacy of the B2 formulation was low throughout the trial, with the exception of month 2 where mosquito mortality was 90%. In contrast, spraying the B3 formulation at a target dose of 100 mg/m^2^ resulted in mortality between 81% and 100% for 5 months in both mosquito strains. The B3 formulation at the highest application rate (target dose 150 mg/m^2^) killed between 87–100% of both strains throughout the 7 months of the trial.

### Efficacy of broflanilide 50WP against free-flying wild mosquitoes entering the huts

Hut entry and exophily. Mosquitoes were collected for two discrete periods in each hut trial: from the end of August to the beginning of October and from January to the end of February. From mid-October to December mosquito densities were so low that collecting wild mosquitoes did not yield sufficient numbers. In total 1048 *Anopheline* mosquitoes were collected during the first hut trial which was conducted for 48 nights between September 2017 and February 2018. 1139 *Anopheline* mosquitoes were collected during the second trial which was conducted for 56 nights between August 2018 and February 2019. In both studies the percentage natural exophily was high, with 100% mosquitoes in the control huts exiting during the night. Due to the high natural exophily it was not possible to determine the insecticide induced exophily.

Blood feeding. Blood feeding success in the first hut trial was 74% in the control treatment and between 57% and 71% in the broflanilide 50WP arms (Table 2). Compared to the untreated control blood feeding was significantly reduced for the pirimiphos methyl treatment and the 50 mg/m^2^ treatments on both concrete and mud. Blood feeding inhibition was 16% (p<0.05) for pirimiphos methyl and 23% (p<0.01) and 18% (p<0.05) respectively for the 50 mg/m^2^ broflanilide 50WP target concentration on concrete and mud.

**Table 2:** Number of free flying *An*. *arabiensis* blood fed and blood feeding inhibition in experimental huts treated with broflanilide 50WP or pirimiphos-methyl (PMM).

Trial	Treatment	Negative Control	Broflanilide 50WP						PMM
2017 hut trial	Substrate	Concrete	Concrete			Mud			Concrete
	Target dose	-	50 mg/m^2^	100 mg/m^2^	200 mg/m^2^	50 mg/m^2^	100 mg/m^2^	200 mg/m^2^	1000 mg/m^2^
	No. collected	155	92	86	261	124	68	163	99
	No. blood fed	114	52	61	175	75	43	107	61
	% blood feeding	73.5^a^	56.5^b^	70.9^a^	67.1^a,b^	60.5^b^	63.2^a,b^	65.6^a,b^	61.6^b^
	95% CI	[66.5–80.6]	[46.2–66.8]	[61.1–80.7]	[61.3–72.8]	[51.9–69.2]	[51.5–75.0]	[58.3–73.0]	[51.9–71.4]
	% BF Inhibition	-	23.1	NS	NS	17.8	NS	NS	16.2
2018 hut trial	Target dose	-	B2 100 mg/m^2^	B3 100 mg/m^2^	B3 150 mg/m^2^	B2 100 mg/m^2^	B3 100 mg/m^2^	B3 150 mg/m^2^	1000 mg/m^2^
	No. collected	101	199	173	112	265	102	121	66
	No. blood fed	28	44	36	16	35	32	26	12
	% blood feeding	27.7^a^	22.1^ac^	20.9^ac^	14.3^bc^	13.3^b^	31.4^a^	21.7^ac^	18.2^ab^
	95% CI	[18.8–36.6]	[16.3–27.9]	[14.8–27.1]	[7.7–20.9]	[9.1–17.4]	[22.2–40.5]	[14.2–29.1]	[8.6–27.7]
	% BF Inhibition	-	NS	NS	48.5	52.4	NS	NS	NS

In the second hut trial blood feeding in the control hut was much lower (28%) yet a significant reduction in blood feeding was still found for two of the treatments; the B3 formulation at 150 mg/m^2^ on concrete and the B2 formulation at 100 mg/m^2^ on mud. Blood feeding inhibition compared to the control treatment was 49% (p<0.05) and 52% (p<0.05) respectively.

#### 72h mortality

Mortality of free flying *An*. *arabiensis* mosquitoes was recorded 72 hours after collection due to the delayed mortality seen with broflanilide (Table 3). In both hut trials mortality in all treatment arms was significantly higher than the control mortality. Three concentrations of the broflanilide 50WP B2 formulation were compared in the first hut trial. The highest application rate of the B2 formulation gave the highest mosquito mortality: 75.5% on concrete and 67.5% on mud. Mortality of mosquitoes exposed to the 200mg/m^2^ broflanilide 50WP B2 application rate was significantly higher than in the hut sprayed with pirimiphos methyl, both on concrete (p<0.001) and on mud (p<0.01). On the concrete surface there was a significant difference between the 200 mg/m^2^ target dose and both the 100 mg/m^2^ and the 50 mg/m^2^ target doses, 75.5% vs 60.5% (p<0.01) and 56.5% mortality (p<0.001). On the mud surface the 200mg/m^2^ broflanilide 50WP B2 application showed significantly higher mortality than the 100 mg/m^2^ application, 67.5% vs 54.4% mortality (p<0.05).

**Table 3:** Mortality of free flying *An*. *arabiensis* 72h after collection in experimental huts treated with broflanilide 50WP or pirimiphos-methyl (PMM).

Trial	Treatment	Negative Control	Broflanilide 50WP						PMM
2017 hut trial	Substrate	Concrete	Concrete			Mud			Concrete
	Target dose	-	50 mg/m^2^	100 mg/m^2^	200 mg/m^2^	50 mg/m^2^	100 mg/m^2^	200 mg/m^2^	1000 mg/m^2^
	No. collected	155	92	86	261	124	68	163	99
	No. dead at 72h	50	52	52	197	74	37	110	48
	%72h mortality	32.3^a^	56.5^b,c^	60.5^b,c^	75.3^d^	59.7^b,c^	54.4^b^	67.5^c,d^	48.5^b^
	95% CI	[24.8–39.7]	[46.2–66.8]	[49.9–71.0]	[70.2–80.7]	[50.9–68.4]	[42.3–66.6]	[60.2–74.8]	[38.5–58.5]
2018 hut trial	Target dose	-	B2 100 mg/m^2^	B3 100 mg/m^2^	B3 150 mg/m^2^	B2 100 mg/m^2^	B3 100 mg/m^2^	B3 150 mg/m^2^	1000 mg/m^2^
	No. collected	101	199	173	112	265	102	121	66
	No dead at 72h	7	100	118	75	97	46	64	28
	%72h mortality	6.9^a^	50.3^b^	68.2^c^	67.0^c^	36.6^d^	45.1^b,d^	52.5^b,d^	42.4^b,d^
	95% CI	[1.9–12.0]	[43.2–57.3]	[61.2–75.2]	[58.1–75.8]	[30.8–42.4]	[35.3–54.9]	[43.4–61.6]	[30.2–54.7]

When comparing the B2 formulation with the B3 formulation in the second hut trial, the latter formulation gave a significantly higher mortality in wild mosquitoes when sprayed at a target concentration of 100 mg/m^2^ on a concrete surface, i.e. 68.2% compared to 50.3% (p<0.01). On the mud surface, although the mortality was higher for the B3 formulation, 44.1% compared to 36.6% for the B2 formulation, the difference in mortality was not was not statistically significant. The B3 formulation sprayed at the higher rate did not result in a significant higher mortality when compared to the 100 mg/m^2^ target dose. The pirimiphos methyl treatment on concrete killed 48.5% of wild free-flying *An*. *arabiensis* in the first hut trial and 42.4% in the second trial.

### Molecular analysis of species identification and *kdr*-status

From each hut trial a subsample of mosquitoes was analysed using PCR to determine the species ID and the presence or absence of the *kdr-e* (L1014S) mutation. In the first hut trial 87 mosquitoes were analysed which all were identified as *Anopheles arabiensis*. The same 87 mosquitoes were used in the *kdr* analysis; 85 mosquitoes were homozygous susceptible (SSe) and 2 mosquitoes did not amplify.

For the second hut trial 100 mosquitoes were analysed, out of which 99 were *Anopheles arabiensis* and 1 did not amplify, even after repeated analysis. All mosquitoes that amplified (99/100) were homozygous *kdr* susceptible for the L1014S mutation.

### Quality of spray application

Liquid samples taken from the spray tank solutions, and filter papers (sprayed from the same tanks) were analysed to determine the quality of the IRS application in the experimental huts. Samples from the spray tank solution indicated that all broflanilide 50WP concentrations were made within ±2% of the target dose, apart from the 100 mg/m^2^ on mud treatment which was 29% under the target dose. The pirimiphos methyl sample taken from the spray tank before spraying was 13% lower than target concentration before spraying and 57% lower after spraying, indicating non-homogeneous mixing in the tank.

The mean concentration of insecticide sprayed on ten filter papers was calculated for each hut (Table 4). The pirimiphos methyl treatment in the second hut trial showed 86% deviation from target dose. Other treatment arms were all within ±50% of the target concentration. In the first hut study the deviation from the target dose ranged between -37% and +32%, whereas in the second hut study treatment arms were generally underdosed, varying from -14% to -42%. These results correspond with the samples taken from the spray solution, which indicate that in the second hut trial the solution in the tank was also less concentrated than the target concentration.

**Table 4:** Mean application rate [%95 CI] of broflanilide 50 WP (BRF) or pirimiphos methyl (PMM) as measured by extractions from filter papers followed by HPLC and percentage deviation from the target rate.

Trial	Hut treatment	Substrate	Active ingredient content (mg/m^2^)	% Deviation
2017 hut trial	BRF 50 mg/m^2^	Concrete	66 [40–92]	32
	BRF 100 mg/m^2^	Concrete	66 [38–94]	-34
	BRF 200 mg/m^2^	Concrete	183 [112–254]	-9
	BRF 50 mg/m^2^	Mud	57 [32–82]	14
	BRF 100 mg/m^2^	Mud	87 [28–146]	-13
	BRF 200 mg/m^2^	Mud	258 [121–396]	29
	PMM 1000 mg/m^2^	Concrete	637 [257–1017]	-37
2018 hut trial	BRF B2 100 mg/m^2^	Concrete	72 [36–108]	-28
	BRF B3 100 mg/m^2^	Concrete	65 [40–91]	-35
	BRF B3 150 mg/m^2^	Concrete	96 [70–123]	-36
	BRF B2 100 mg/m^2^	Mud	58 [29–86]	-42
	BRF B3 100 mg/m^2^	Mud	86 [35–138]	-14
	BRF B3 150 mg/m^2^	Mud	90 [54–126]	-40
	PMM 1000 mg/m^2^	Concrete	136 [85–186]	-86

## Discussion

New chemistry for vector control is urgently needed. The development and optimisation of a new insecticide in a malaria endemic country under realistic conditions is crucial to establish its potential for vector control. Here the novel active ingredient broflanilide (tradename TENEBENAL^TM^) was tested for the first time in an experimental hut setting in Tanzania. Two hut trials were conducted, the first one to evaluate three different application rates of a prototype broflanilide 50WP formulation, B2, and the second one to evaluate an improved formulation, B3. The study was designed to determine a formulation and application rate of broflanilide that has long-lasting residual efficacy on both mud and concrete surfaces.

In both hut trials, the broflanilide 50WP formulations were shown to be effective against both the insectary-reared insecticide susceptible Kisumu strain and the pyrethroid-resistant Muleba-Kis which has a fixed L1014S mutation and elevated P450s. The *kdr* mutation reduces the susceptibility of mosquitoes to both DDT and pyrethroids, and has been reported in 61 of the 76 malaria endemic countries [33]. The absence of cross-resistance between broflanilide and the pyrethroid resistance mechanisms (as shown in the present paper) is essential for the future utility of broflanilide IRS in the rotation of IRS products for insecticide resistance management and the control of malaria transmitted by pyrethroid resistant vectors. Residual efficacy of broflanilide is dose dependent; results in the first hut study showed that the lowest application rate showed a duration of effective action of 3 months against Kisumu and 2 months against the Muleba-Kis strain on a mud surface whereas the highest application rate remained efficacious for the full duration of the trial on both the mud and the concrete surface.

An improved formulation was developed to increase performance on mud surfaces at low(er) concentrations. This B3 formulation showed the same residual efficacy as the B2 formulation on a concrete substrate but had a prolonged efficacy on mud, increasing the duration of effective action from 1 month to over 5 months for the Kisumu strain and from less than 1 month to more than 6 months for the Muleba-Kis strain. When comparing the B2 and the B3 formulation directly we cannot rule out that the limited effect on mosquitoes for the B2 formulation was due to underdosing rather than formulation. Yet the B2 formulation in the 2017 trial was sprayed at a similar rate as the B3 arm in 2018 and still gave poor performance on mud. Whilst it is important to acknowledge that the treatments in the second trial were all underdosed, the results were nevertheless very promising. The B3 at 100 mg/m^2^ and 150 mg/m^2^ treatments gave at least 6 months residual efficacy, despite the lower a.i. contents as determined by filter papers of 86 and 90 mg/m^2^ respectively. Reflecting these data, it is appropriate to conclude that an application rate of 100 mg/m^2^ will give sufficient duration of effective action to provide protection lasting an entire malaria transmission season.

It is anticipated that the mortality of wild free-flying *An*. *arabiensis* is lower compared to the mortality of the insectary-reared strains, as the exposure to the treated surface differs from the time and movement-controlled exposure in *in situ* cones. *An*. *arabiensis* shows strong exophilic behaviour and thus may not rest for prolonged period on the insecticide-treated surfaces. Regardless, broflanilide also showed good residual efficacy against the wild free-flying *An*. *arabiensis* mosquitoes in lower Moshi. Mortality in the first trial was as high as 76% on concrete and 68% on mud, although noting that the control mortality was higher than expected. In the second trial the control mortality was <10%, and broflanilide exposure resulted in 68% mortality with the 100 mg/m^2^ application rate on concrete and 53% on the mud surface. The reference treatment, pirimiphos-methyl (Actellic® 300CS) was equivalent or showed a significantly lower mortality compared to the broflanilide treatments. Previous hut trials with pirimiphos-methyl reported mortality levels against wild *An*. *arabiensis* mosquitoes of around 80% after 6 months on a concrete substrate [10]. The shorter residual efficacy of the pirimiphos-methyl treatment in the second trial is likely due to the underdosing of the treatment.

The *An*. *arabiensis* population in lower Moshi was previously found to have elevated levels of P450 mixed function oxidases and β-esterases [24, 34]. Metabolic resistance is thought to play a major role in resistance against multiple insecticide classes in Sub-Saharan Africa [35]. Recent CDC bottle bioassays indicated restored susceptibility after pre-exposure to PBO, and resistance to 5x the diagnostic dose of alphacypermethrin (unpublished data). The ability of broflanilide 50 WP to successfully control this metabolic-resistant wild vector population is an important indication that this novel insecticide will make a valuable contribution to malaria control alongside other long-lasting public health insecticides.

Between 2009 and 2013 many IRS campaigns shifted from using pyrethroids products to using alternative insecticides to counter malaria being transmitted by pyrethroid resistant vectors. New insecticide classes that are effective against pyrethroid-resistant mosquito populations will be needed to tackle and put out flares of transmission [36] that will inevitably arise in the march towards elimination. In the current situation with a high coverage of pyrethroid-nets the continued use of IRS is essential, either in high transmission areas to bring down transmission to the point so that other forms of malaria vector control such as dual-a.i long-lasting nets can be used to greater effect [37], or in low-transmission areas in a reactive focal malaria intervention strategy [38].

The B3 formulation of broflanilide 50WP sprayed at a target concentration of 100 mg a.i./m^2^ has shown encouraging residual efficacy in this study and this application rate would be suitable for evaluation in a large-scale setting. Community studies with this product, which has now been branded VECTRON^TM^ T500, are planned in Benin and Tanzania and will commence in 2021. This novel insecticidal product has potential as a promising new IRS product for use in rotations with IRS formulations containing insecticides with different modes of action in insecticide resistance management programmes.

## Conclusion

IRS programmes are based on the premise that the IRS products have a duration of effective action sufficient to cover the malaria transmission season, i.e. 6 months or longer. Mosquito mortality in huts sprayed with broflanilide 50WP formulations remained high 5–6 months post-spraying in both hut trials, except for the mud surface in the first hut study. It was shown that broflanilide is effective against both susceptible and pyrethroid-resistant mosquito strains, and thus could potentially be used for malaria transmission control in a setting where the malaria vectors are highly pyrethroid resistant. The B3 formulation of broflanilide 50WP, brand name VECTRON^TM^ T500, sprayed at a target concentration of 100 mg a.i. /m^2^ is recommended for further evaluation in a community setting.

## References

[1] Cibulskis RE, Alonso P, Aponte J, Aregawi M, Barrette A, Bergeron L, et al. Malaria: Global progress 2000–2015 and future challenges. Infect Dis Poverty. 2016;5(1):61. 10.1186/s40249-016-0151-8 27282148 PMC4901420

[2] Bhatt S, Weiss D, Cameron E, Bisanzio D, Mappin B, Dalrymple U. The effect of malaria control on Plasmodium falciparum in Africa between 2000 and 2015. Nature. 2015;526. 10.1038/nature15535 26375008 PMC4820050

[3] WHO. World malaria report. Geneva: WHO, 2019.

[4] Killeen GF, Ranson H. Insecticide-resistant malaria vectors must be tackled. Lancet. 2018. 10.1016/S0140-6736(18)30844-4 . 29655495

[5] Hemingway J. The role of vector control in stopping the transmission of malaria: threats and opportunities. Philosophical Transactions of the Royal Society of London—Series B: Biological Sciences. 2014;369(1645):20130431. 10.1098/rstb.2013.0431 . 24821917 PMC4024224

[6] WHO. Guidelines for malaria vector control. Geneva: WHO, 2019. 30844152

[7] WHO. Global plan for insecticide resistance management in malaria vectors. Geneva: WHO, 2012. 10.1186/s12936-015-0693-4 PMC4423491 25899397

[8] Hemingway J, Ranson H, Magill A, Kolaczinski J, Fornadel C, Gimnig J. Averting a malaria disaster: will insecticide resistance derail malaria control? Lancet. 2016;387. 10.1016/S0140-6736(15)00417-1 26880124 PMC6215693

[9] Roberts L, Enserink M. Malaria. Did they really say… eradication? Science. 2007;318(5856):1544–5. 10.1126/science.318.5856.1544 . 18063766

[10] Oxborough RM, Kitau J, Jones R, Feston E, Matowo J, Mosha FW, et al. Long-lasting control of Anopheles arabiensis by a single spray application of micro-encapsulated pirimiphos-methyl (Actellic 300 CS). Malaria Journal. 2014;13:37. 10.1186/1475-2875-13-37 . 24476070 PMC3914366

[11] WHOPES. Report of the Sixteenth WHOPES Working Group Meeting. Geneva: WHO, 2013.

[12] WHO. WHO prequalification Vector Control [cited 2020 04 Jul]. Available from: https://www.who.int/pq-vector-control/prequalified-lists/en/.

[13] Katsuta H, Nomura M, Wakita T, Daido H, Kobayashi Y, Kawahara A, et al. Discovery of broflanilide, a novel insecticide. J Pestic Sci. 2019;44(2):120–8. 10.1584/jpestics.D18-088 . 31148938 PMC6529746

[14] Lees RS, Ambrose P, Williams J, Morgan J, Praulins G, Ingham VA, et al. Tenebenal: a meta-diamide with potential for use as a novel mode of action insecticide for public health. Malar J. 2020;19(1):398. Epub 2020/11/11. 10.1186/s12936-020-03466-4 . 33168015 PMC7654575

[15] Nakao T, Banba S. Broflanilide: A meta-diamide insecticide with a novel mode of action. Bioorganic & Medicinal Chemistry. 2016;24(3):372–7. 10.1016/j.bmc.2015.08.008 26361738

[16] IRAC. IRAC Mode of Action Classification Scheme. Version 9.32019 January 2020; Version 9.3.

[17] Rowland M, Boko P, Odjo A, Asidi A, Akogbeto M, N’Guessan R. A new long-lasting indoor residual formulation of the organophosphate insecticide pirimiphos methyl for prolonged control of pyrethroid-resistant mosquitoes: an experimental hut trial in Benin. PLoS ONE [Electronic Resource]. 2013;8(7):e69516. 10.1371/journal.pone.0069516 . 23936033 PMC3720653

[18] Oxborough RM, Kitau J, Jones R, Mosha FW, Rowland MW. Experimental hut and bioassay evaluation of the residual activity of a polymer-enhanced suspension concentrate (SC-PE) formulation of deltamethrin for IRS use in the control of Anopheles arabiensis. Parasites & Vectors [Electronic Resource]. 2014;7:454. 10.1186/s13071-014-0454-1 . 25274145 PMC4189627

[19] IVCC. Indoor Residual Spray—Target Product Profile 2018 [cited 2020 04 Jul]. Available from: https://www.ivcc.com/research-development/target-product-profiles-tpps/.

[20] Bradley J, Matias A, Schwabe C, Vargas D, Monti F, Nseng G, et al. Increased risks of malaria due to limited residual life of insecticide and outdoor biting versus protection by combined use of nets and indoor residual spraying on Bioko Island, Equatorial Guinea. Malaria Journal. 2012;11(1):242. 10.1186/1475-2875-11-242 22835049 PMC3458978

[21] Ijumba JN, Mosha FW, Lindsay SW. Malaria transmission risk variations derived from different agricultural practices in an irrigated area of northern Tanzania. Medical and Veterinary Entomology. 2002;16(1):28–38. 10.1046/j.0269-283x.2002.00337.x 11963979

[22] Protopopoff N, Matowo J, Malima R, Kavishe R, Kaaya R, Wright A, et al. High level of resistance in the mosquito Anopheles gambiae to pyrethroid insecticides and reduced susceptibility to bendiocarb in north-western Tanzania. Malaria Journal. 2013;12:149. 10.1186/1475-2875-12-149 . 23638757 PMC3655935

[23] WHO. Test procedures for insecticide resistance monitoring in malaria vector mosquitoes. Second edition. Geneva2018.

[24] Matowo J, Jones CM, Kabula B, Ranson H, Steen K, Mosha F, et al. Genetic basis of pyrethroid resistance in a population of Anopheles arabiensis, the primary malaria vector in Lower Moshi, north-eastern Tanzania. Parasites & Vectors [Electronic Resource]. 2014;7:274. 10.1186/1756-3305-7-274 . 24946780 PMC4082164

[25] Smith A. A verandah-trap hut for studying the house-frequenting habits of mosquitos and for assessing insecticides. I. A description of the verandah-trap hut and of studies on the egress of Anopheles gambiae Giles and Mansonia uniformis (Theo.) from an untreated hut. Bull Entomol Res. 1964;56. 10.1017/s0007485300057114 5831207

[26] Massue DJ, Kisinza WN, Malongo BB, Mgaya CS, Bradley J, Moore JD, et al. Comparative performance of three experimental hut designs for measuring malaria vector responses to insecticides in Tanzania. Malaria Journal. 2016;15:165. 10.1186/s12936-016-1221-x PMC4793500. 26979404 PMC4793500

[27] Oxborough RM, Kitau J, Mosha FW, Rowland MW. Modified veranda-trap hut for improved evaluation of vector control interventions. Medical and Veterinary Entomology. 2015;29(4):371–9. 10.1111/mve.12123 26194052

[28] WHO. Manual for Indoor Residual Spraying. Application of residual sprays for vector control. 2007.

[29] WHO. Guidelines for testing mosquito alduticides for indoor residual spraying and treatment of mosquito nets. Geneva: World Health Organization, 2006.

[30] Kweka EJ, Mahande AM. Comparative evaluation of four mosquitoes sampling methods in rice irrigation schemes of lower Moshi, northern Tanzania. Malar J. 2009;8:149. 10.1186/1475-2875-8-149 19580663 PMC2712478

[31] Bass C, Williamson MS, Wilding CS, Donnelly MJ, Field LM. Identification of the main malaria vectors in the Anopheles gambiae species complex using a TaqMan real-time PCR assay. Malar J. 2007;6:155. Epub 2007/11/24. 10.1186/1475-2875-6-155 18034887 PMC2213665

[32] Bass C, Nikou D, Donnelly MJ, Williamson MS, Ranson H, Ball A, et al. Detection of knockdown resistance (kdr) mutations in Anopheles gambiae: a comparison of two new high-throughput assays with existing methods. Malar J. 2007;6:111. Epub 2007/08/19. 10.1186/1475-2875-6-111 17697325 PMC1971715

[33] WHO. World malaria report Geneva: WHO, 2017.

[34] Matowo J, Kulkarni MA, Mosha FW, Oxborough RM, Kitau JA, Tenu F, et al. Biochemical basis of permethrin resistance in Anopheles arabiensis from Lower Moshi, north-eastern Tanzania. Malaria Journal. 2010;9:193. 10.1186/1475-2875-9-193 . 20609220 PMC3224900

[35] Ranson H, N’Guessan R, Lines J, Moiroux N, Nkuni Z, Corbel V. Pyrethroid resistance in African anopheline mosquitoes: what are the implications for malaria control? Trends Parasitol. 2011;27. 10.1016/j.pt.2010.08.004 20843745

[36] Oxborough RM. Trends in US President’s Malaria Initiative-funded indoor residual spray coverage and insecticide choice in sub-Saharan Africa (2008–2015): urgent need for affordable, long-lasting insecticides. Malaria Journal. 2016;15:146. 10.1186/s12936-016-1201-1 . 26957210 PMC4784374

[37] Protopopoff N, Mosha JF, Lukole E, Charlwood JD, Wright A, Mwalimu CD, et al. Effectiveness of a long-lasting piperonyl butoxide-treated insecticidal net and indoor residual spray interventions, separately and together, against malaria transmitted by pyrethroid-resistant mosquitoes: a cluster, randomised controlled, two-by-two factorial design trial. Lancet. 2018. 10.1016/S0140-6736(18)30427-6 . 29655496 PMC5910376

[38] Hsiang MS, Ntuku H, Roberts KW, Dufour MK, Whittemore B, Tambo M, et al. Effectiveness of reactive focal mass drug administration and reactive focal vector control to reduce malaria transmission in the low malaria-endemic setting of Namibia: a cluster-randomised controlled, open-label, two-by-two factorial design trial. Lancet. 2020;395(10233):1361–73. Epub 2020/04/27. 10.1016/S0140-6736(20)30470-0 32334702 PMC7184675

